# Typical symptoms and not positive reflux-cough correlation predict cure of gastroesophageal reflux disease related chronic cough after laparoscopic fundoplication: a retrospective study

**DOI:** 10.1186/s12876-019-1027-8

**Published:** 2019-06-26

**Authors:** Dong Chen, Zhonggao Wang, Zhiwei Hu, Yan Liang, Fei Xiao, Jimin Wu

**Affiliations:** 1 Department of vascular surgery, xuanwu hospital, Capital medical unversity, Beijing, China; 2 Department of gastroesophageal reflux disease, The General Hospital of the PLA Rocket Force, Beijing, China

**Keywords:** Gastroesophageal reflux disease, Cough, Fundoplication

## Abstract

**Background:**

The effect of laparoscopic fundoplication on reflux-related chronic cough is unpredictable, the aim of the study is to investigate the predictive effect of positive reflux-cough correlation on the resolution of reflux-related chronic cough after anti-reflux surgery.

**Methods:**

A 5 years retrospective review was performed. Logistic regression analysis was used to determine the independent predictors on the cure of chronic cough.

**Results:**

Seventy-nine patients were included in this study, among which chronic cough was cured in 47 (59.5%) and significantly improved in 10 (12.7%) patients. Present of typical symptoms (odds ratio = 6.435,95% confidence interval [CI] = 1.427–29.032, *p* = 0.015) and number of Reflux episodes (impedance) ≥73 (odds ratio = 0.306, 95% confidence interval [CI] = 0.107–0.874, *p* = 0.027) were significantly associated with the cure of chronic cough.

**Conclusions:**

laparoscopic fundoplicaiton is effective for the management of reflux-related chronic cough, particularly with the present of typical symptoms.

**Trial registration:**

(Trial registration number: ChiCTR1800016444; Trial registration date: June 01, 2018)

## Background

Gastroesophageal reflux disease (GERD) is a condition which develops when the reflux of stomach contents causes troublesome symptoms and/or complications [1, 2]. The typical symptoms of GERD are heartburn and regurgitation, while the atypical symptoms of GERD include non-cardiac chest pain and extraesophageal symptoms such as chronic cough, chronic asthma, chronic laryngitis and dental erosions [3–5]. The prevalence of GERD in East Asia is about 2.5–7.8% [6]. Chronic cough defined as cough that persisits for > 8 weeks, affects 11–20% of the adult population and significantly impairs health-related quality of life [7]. GERD symptom response rates for anti-reflux surgery varies based on 1) evidence for GERD, such as ambulatory pH monitoring metrics, PPI response and GERD phenotypes, and 2) the particular symptom [3]. while laproscopic fundoplication (LF) can control typical symptoms in about 90% of patients [8–13], the resolution of chronic cough is less predictive (about 51–96%) [9, 10, 14, 15]. Impedance-pH monitoring is widely used in the diagnosis of GERD, and its ability to simultaneously record symptoms allows evaluation of reflux-symptom correlation [16]. The aim of this study was to investgate if positive reflux-cough correlation predicts resolution of chronic cough after antireflux surgery.

## Methods

### Study population

We retrospectively analysis information from patients who underwent LF in The General Hospital of the PLA Rocket Force from January 2013 to December 2017. Inclusion criteria (all of the two): chronic cough persists for more than 8 weeks; complete 24-h multichannel intraluminal impedance pH (24-h MII-pH) monitoring and at least one cough was recorded. Exclusion criteria [17] (at least one): present smoker; use of angiotensin-converting-enzyme inhibitors (ACEI); abnormal chest radiograph; recently upper respiratory infection; upper airway cough syndrome; previous upper gastrointestinal surgery; malignancy; major disorders of peristalsis (absent contractility, distal esophageal spasm, and jackhammer esophagus). The following information was collected form each patient eligible for this study: demographics factors (gender, age, body mass index (BMI), hypertension, diabetes and coronary heart disease), symptoms (heartburn, regurgitation, chronic cough, chronic asthma), response of chronic cough to proton pump inhibitor (PPI), hiatal hernia presence (endoscopic and/or high resolution manometry), endoscopic factors (esophagitis, barrett’s esophagus, reflux of bile), impedance-pH monitoring factors [DeMeester score, acid exposure time (AET), bolus exposure time (BET), symptom index (SI), symptom association probability (SAP)] and high resolution manometry (HRM) determined ineffective esophageal motility (50% or more ineffective esophageal swallows).

### 24-h MII-pH monitoring

All reflux events were collected from pH-impedance monitoring, and all impedance-pH monitoring were performed off PPI for at least 7 days as previously described [18]. Symptoms were considered related to reflux events if they occurred within 2 min after the reflux event. The symptom index (SI) was defined as the ratio of reflux-related symptoms to the total number of symptoms [19], the sympton-association probability was calculated as Weusten [20] described. Positive reflux-cough correlation defined as SI > 50% and/or SAP > 95%.

### Outcome

Eligible subjects were contacted for a telephone interview to determine symptomatic outcome of chronic cough and typical symptoms. Symptomatic outcome was considerd cured if the patient was symptom-free or minor complaints remained but required no medication (PPI, H2 receptor antagonists, prokinetic agents, antacids and antitussive); excellent if symptoms were significantly improved but required on-demand medication; fair if reflux symptoms were slightly improved and required daily dose of medication; poor if the symptoms were not improved and required more than daily dose of medication or refractory to medical therapy.

### Statistical analysis

All statistical tests were performed using IBM SPSS Statistics (Version 20). The chi-square test or Fisher’s exact test was performed to evaluate univariate effects of each predictor variable on outcome (chronic cough cured or not cured). A stepwise forward logistic regression was performed to determine independent predictors of outcome (chronic cough cured or not cured). statistical significance was considered at the *p* < 0.05 level.

## Results

**Fig. 1:**
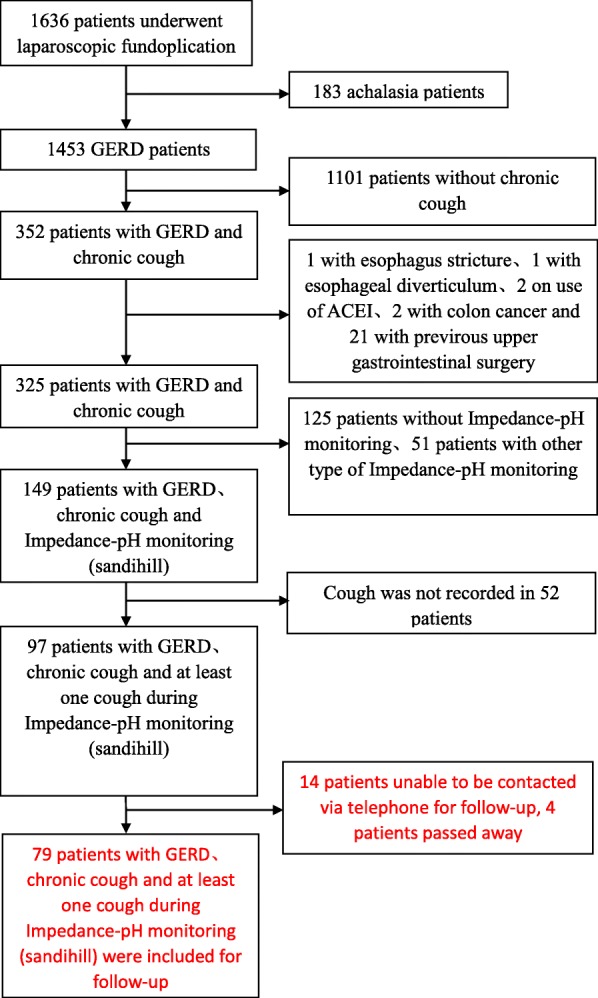
Study flow diagram

Between January 2013 and December 2017, a total of 1636 patients underwent laparoscopic fundoplication, among which 97 patients meet the criteria for further follow up (Fig. 1). Fourteen patients couldn’t be reached and 4 patients died (1 for leukemia, 1 for pulmonary fibrosis and two unclear), thus 79 patients were included in this study. Among which 30 patients underwent Nissen-Rossetti procedure, 34 underwent Nissen procedure, 14 under Toupet procedure, and 1 underwent Dor procedure.

The demographics factors and symptomatic outcomes among the 79 patients were listed in Table 1 and Table 2, respectively. At a median follow-up time of 35 months after surgery (range 7–65 months), symptomatic outcome of chronic cough was rated cured in 47 (59.5%) and excellent in 10 (12.7%) of the 79 patients. Heartburn was rated cured in 47 (79.7%) and excellent in 5 (8.5%) of the 59 patients. Regurgitation was rated cured in 46 (74.2%) and excellent in 7 (11.3%) of the 62 patients.

**Table 1:** Demographics factors of the study population (*N* = 79)

Median age (year)	60(range14–79)
Male	43 (54.4%)
Body mass index	
normal(18.5–24.99)	43 (54.4%)
overweight(25–29.99)	30 (38.0%)
obesity(≥30)	6 (7.6%)
Hypertension	20 (25.3%)
Diabetes	3 (3.8%)
Coronary heart disease	3 (3.8%)

**Table 2:** symptomatic outcomes among 79 patients

	Cured	Excellent	Fair	Poor	Total
Chronic cough	47 (59.5%)	10 (12.7%)	4 (5.1%)	18 (22.7%)	79
Heartburn	47 (79.7%)	5 (8.5%)	1 (1.7%)	6 (10.1%)	59
Regurgitation	46 (74.2%)	7 (11.3%)	2 (3.2%)	7 (11.3%)	62
Asthma	9 (39.1%)	4 (17.4%)	7 (30.4%)	3 (13%)	23

At the univariate level (Table 3), three factors were significantly associated with the cure rate of chronic cough. These included typical symptoms, Number of Reflux episodes (impedance) and Ineffective esophageal motility. At the multivariate level, all the factors listed in Table 3 together with BMI (overweight, obesity vs normal) and type of surgery (Nissen-Rossetti, Nissen vs Toupet) were offered to the regression procedure. Present of typical symptoms (OR = 6.435, 95%CI = 1.427–29.032, *p* = 0.015) and number of Reflux episodes (impedance) ≥73 (OR = 0.306, 95%CI = 0.107–0.874, *p* = 0.027) were independently associated with the cure of chronic cough (Table 4). we also performed the same analysis procedure on cure+excellent vs fair+poor and cure vs fair+poor of chronic cough, and found that only present of typical symptoms was the independent predictor (OR = 4.114, 95%CI = 1.091–15.522, *p* = 0.037; OR = 5.857, 95%CI = 1.290–26.590, *p* = 0.022).

**Table 3:** Univariate association with outcome

Variable	Outcome		*P* value
	Cured	Not cured	
Age			
≥ 60	25 (61%)	16 (39%)	
< 60	22 (57.9%)	16 (42.1%)	0.78
Sex			
Male	25 (58.1%)	18 (41.9%)	
Femal	22 (61.1%)	14 (38.9%)	0.789
Hypertension			
Yes	11 (55%)	9 (45%)	
No	36 (61%)	23 (39%)	0.636
Diabetes			
Yes	3 (100%)	0 (0%)	
No	44 (57.9%)	32 (42.1%)	0.391
Coronary heart disease			
Yes	1 (33.3%)	2 (66.7%)	
No	46 (60.5%)	30 (39.5%)	0.733
Heartburn			
Yes	38 (64.4%)	21 (35.6%)	
No	9 (45.0%)	11 (55.0%)	0.127
Regurgitation			
Yes	40 (64.5%)	22 (35.5%)	
No	7 (41.2%)	10 (58.8%)	0.082
Typical symptoms			
Yes	44 (64.7%)	24 (35.3%)	
No	3 (27.3%)	8 (72.7%)	0.044
Asthma			
Yes	17 (73.9%)	6 (26.1%)	
No	30 (53.6%)	26 (46.4%)	0.094
Hiatal hernia			
Yes	35 (60.3%)	23 (39.7%)	
No	12 (57.1%)	9 (42.9%)	0.798
Esophagitis			
Yes	21 (56.8%)	16 (43.2%)	
No	26 (61.9%)	16 (38.1%)	0.642
Barrett’s esophagus			
Yes	7 (77.8%)	2 (22.2%)	
No	40 (57.1%)	30 (42.9%)	0.409
Reflux of bile			
Yes	6 (40%)	9 (60%)	
No	41 (64.1%)	23 (35.9%)	0.088
DeMeester scores			
≥ 14.7	22 (55%)	18 (45%)	
< 14.7	25 (64.1%)	14 (35.9%)	0.41
Acid exposure time			
≥ 4.2%	22 (56.4%)	17 (43.6%)	
< 4.2%	25 (62.5%)	15 (37.5%)	0.581
Bolus exposure time			
≥ 1.4%	29 (53.7%)	25 (46.3%)	
< 1.4%	18 (72%)	7 (28%)	0.123
Number of Reflux episodes (impedance)			
≥ 73	18 (47.4%)	20 (52.6%)	
< 73	29 (70.7%)	12 (29.3%)	0.035
SI of chronic cough			
> 50%	17 (58.6%)	12 (41.4%)	
≤ 50%	30 (60%)	20 (40%)	0.904
SAP of chronic cough			
> 95%	22 (56.4%)	17 (43.6%)	
≤ 95%	25 (62.5%)	15 (37.5%)	0.581
Reflux-cough correlation			
Positive	26 (56.5%)	20 (43.5%)	
Negative	21 (63.6%)	12 (36.4%)	0.525
Ineffective esophageal motility			
Yes	9 (40.9%)	13 (59.1%)	
No	36 (69.2%)	16 (30.8%)	0.023

**Table 4:** Stepwise logistic regression resluts

predictor	Adjusted odds ratio (95% confidence interval)	*P* value
Typical symptoms		
Yes	6.435 (1.427–29.032)	0.015
No	–	–
Number of Reflux episodes (impedance)		
≥ 73	0.306 (0.107–0.874)	0.027
<73	–	–

Among the 79 patients, 19 patients didn’t have pathologic AET and positive reflux-cough association. Fourteen of which had their cough cured or significantly improved (Table 5).

**Table 5:** Detailed information of 19 patients without pathologic AET and positive reflux-cough association

Hiatal hernia	Typical symptoms	No. of reflux episodes ≥73	DeMeester scores ≥14.7	No response to PPI	Partial response to PPI	Complete response to PPI	No use of PPI	No. of patients	Cough cured or significantly improved
yes	yes				yes			5	5
yes	yes					yes		2	2
yes	yes			yes				1	1
yes	yes						yes	1	1
yes	yes	yes			yes			1	0
yes	yes	yes					yes	1	1
yes					yes			1	0
yes							yes	1	0
	yes	yes			yes			1	1
	yes	yes					yes	1	1
	yes				yes			1	1
	yes						yes	2	0
yes	yes		yes	yes				1	0

## Discussion

Seventy-nine patients with chronic cough under LF were included in our study, At a median follow-up time of 35 months after surgery, chronic cough, heartburn and regurgitation were cured or significantly improved in 57(72.2%), 52(88.1%) and 53(85.5%) patients. By using multivariate model, we found that present of typical symptoms (OR = 6.435) and number of Reflux episodes (impedance) ≥73 (OR = 0.306) but not AET and reflux-cough association were independently associated with the cure of chronic cough. GERD, Asthma, and Postnasal drip syndrome, alone or in combination, were responsible for 93.6% of the cases of chronic cough [21, 22]. The 2016 ACCP guidelines recommended a clinical profile to excluding other potential chronic cough causes, and the clinical profile was estimated to be 91% predicive that a patient’s cough would respond to anti-reflux treatment [23]. In our study, asthma and non-asthmatic eosinophilic bronchitis were not excluded, so the respond rate was lower (72.2%).

Francis [24] found that preoperative heartburn with or without regurgitation and esophageal acid exposure of greater than 12% at baseline were significant predictors of response of the primary extraesophageal reflux symptom to anti-reflux surgery. In our study, present of typical symptoms was consistently significantly associated with good outcome in three different multivariate analysis. Number of Reflux episodes (impedance) ≥ 73 was only significantly associated with the cure of chronic cough. Thus we believed that typical symptoms was the only predictor in our study. We utilized a favorable response (cure+excellent) to LF as the “gold standard” test for comparison with present of typical symptoms (consistency test) and found that the consistency was poor (κ value:0.219, *p* = 0.033), thus although typical symptoms can predict better outcome, its ability to diagnose GERD-related cough is poor.

The recent Lyon consensus on ambulatory reflux monitoring illustrated that SI and SAP have a predictive value for the effect of medical and surgical treatment of reflux disease, and this is independent of AET [2]. While data about predictive role of SI and SAP on outcome of LF for chronic cough is still limited and controversial. Marco [9] found that even single time of correlation between cough and reflux could improve cure rate of chronic cough, but the sample size was too small (8 patients in one group and 10 in the other). Michael [25] found that positive SAP independently predicted good outcome of chronic cough, but most patients were under PPI therapy and only pH mornitoring was used. In contrary, Francis [24] found that no difference in response to surgery based on SI/SAP parameters, but the sample size was small too (12 cough patients). Thus our study is basically the first one to investigate the role of reflux-cough correlation on outcome of chronic cough after anti-reflux surgery. We found that positive SI/SAP was not significantly associated with the cure rate of chronic cough. The following two studies may provide a perspective on the reason. Paterson and Murat [26] showed that using a diary or event marker for determination of cough is inadequate because patients underestimate the frequency of cough events or misreport their timing. Sifrim and Dupont [27] found that the number of cough bursts detected by manometry was significantly higher than by patients using the event marker, and the delay was 28 (7–80) seconds, most of all, they found that the delay could change cough-reflux pattern to reflux-cough pattern. Thus correctly calculation of SI/SAP was impossible, not to mention using them as predictive factor for better outcome of LF.

DeMeester scores and AET were also not significantly associated with good outcome of chronic cough. The reason may be that pathologic AET are not the only implication for considering LF. In our center, other evidence of GERD such as hiatal hernia, typical symptoms, No. of reflux episodes, DeMeester scores and PPI response are under consideration. Thus symptomatic outcome of patients without pathological AET may similar to those with pathological AET. As we can see that 14 of 19 patients without pathologic AET and positive reflux-cough association had their cough cured or significantly improved after LF.

There were several limitations in our study. First, as the dose of PPI, duration of PPI, and response of chronic cough to PPI was not standardized in our medical record, thus PPI response was not included. Since a positive response to initial empiric PPI therapy is the best indicator for eventual resolution of gastroesophageal reflux-related chronic cough [28, 29], thus the lack of this part may have an impact on the results. Further, this is a retrospective study and the symptomatic outcome were relied on telephone interview, the patients may misunderstood what we asked, as the cure of chronic cough was easy to judge, thus we adopted cure of chronic cough as the outcome metrics to minimize the impact.

## Conclusion

The clinical profile to excluding other potential chronic cough causes is useful in selecting patients most like to benefit from LF, and present of typical symptoms but not postitive reflux-cough correlation predicts cure of chronic cough. Futher prospective study using manometry to detect cough is needed to illustrate the association between reflux-cough correlation and symptomatic outcome of chronic cough after LF.

## Data Availability

The datasets used and/or analysed during the current study are available from the corresponding author on reasonable request.

## Abbreviations

24-h MII-pH monitoring: 24-h multichannel intraluminal impedance pH monitoring
ACEI: Angiotensin-converting-enzyme inhibitors
AET: Acid exposure time
BET: Bolus exposure time
BMI: Body mass index
GERD: Gastroesophageal reflux disease
HRM: High resolution manometry
LF: Laproscopic fundoplication
PPI: Proton pump inhibitor
SAP: Symptom association probability
SI: Symptom index
